# Advancing medicine one research note at a time: the educational value in clinical case reports

**DOI:** 10.1186/1756-0500-5-293

**Published:** 2012-07-06

**Authors:** Alberto J Cabán-Martinez, Wilfredo F García Beltrán

**Affiliations:** 1 Department of Epidemiology and Public Health, University of Miami, Miller School of Medicine, Miami, FL, USA; 2 Harvard Medical School, Boston, MA, USA; 3 Environmental and Occupational Medicine & Epidemiology, Department of Environmental Health, Harvard University, School of Public Health, 401 Park Drive, Landmark Center Building, 3 Floor, East #49, Boston, MA, 02215, USA

## Abstract

A case report—a brief written note that describes unique aspects of a clinical case—provides a significant function in medicine given its rapid, succinct, and educational contributions to scientific literature and clinical practice. Despite the growth of, and emphasis on, randomized clinical trials and evidenced-based medicine, case reports continue to provide novel and exceptional knowledge in medical education. The journal *BMC Research Notes* introduces a new “case reports” section to provide the busy clinician with a forum in which to document any authentic clinical case that provide educational value to current clinical practice. The aim is for this article type to be reviewed, wherever possible, by specialized Associate Editors for the journal, in order to provide rapid but thorough decision making. New ideas often garnered by and documented in case reports will support the advancement of medical science — one research note at a time.

## Background

Documenting medical case histories to advance the knowledgebase of clinical medicine has long been occurring since the time of Hippocrates (460 B.C.–370 B.C.), and even arguably further back since the papyrus records of ancient Egyptian medicine (c. 1600 B.C.) [1]. These case histories were disseminated in professional medical societies and provided physicians with a written account on how a patient was evaluated for an illness and what could be an expected prognosis for the patient’s particular disease [1]. These case histories—contemporarily called “case reports”—have held a significant function in medicine given their rapid, succinct, and educational contribution to scientific literature and clinical practice [2,3]. Sir William Osler, known by many as the Father of modern medicine, once stated that physicians should “always note and record the unusual…Publish it. Place it on permanent record as a short, concise note. Such communications are always of value” [4]. Over time, this quote has remained valid as case reports continue to foster an educational medium in disseminating new and rare diseases; informing the evaluation, diagnosis, treatment, and prognosis of diseases; and even improving many aspects of medical education.

In the advent of randomized clinical trials (RCTs) and evidenced-based medicine, case reports remain a unique and novel way to communicate anecdotal evidence and brief notes on patient care [5]. In today’s scientific and medical professions, emphasis on publishing clear evidence for the diagnosis and management of illness and diseases using clinical trials is paramount. However, using research methodologies such as RCTs that are costly and time intensive often limit scientists and clinicians to examining few variables and subvert the richness of understanding the influences of patient history, individual characteristics, and disease presentation on disease treatment and outcomes. Case reports often shed light on new diseases, anomalous patient variations, unexpected effects of drugs and their forms of administration, and even different approaches to treating common medical conditions in a relatively quick and inexpensive format [6].

*BMC Research Notes* was set up with the unique aim of reducing the losses suffered by the research and medical community when results remain unpublished because they do not form a sufficiently complete story to justify the publication of a full research article. In keeping with this inclusive ethos, it is felt that everyday cases, as well as particularly rare ones, have significant educational value. Consequently, *BMC Research Notes* introduces a new “case report” article type to provide the busy clinician with a forum in which to document any authentic clinical case that provides educational value to current clinical practice.

## Educational value in case reports

Case reports are an excellent tool for sharing educational experiences related to clinical practice. From the evaluation of a patient’s history and physical examination, to thorough consideration of a differential diagnosis, selection of a treatment plan, and outcomes of different treatment modalities, brief notes in the form of a case report format provide a mechanism in which to document and share these experiences. This becomes indispensable in instances where, for example, there is an unanticipated yet informative outcome or adverse side effect observed in the course of treating a patient or from one who has an anomalous presentation or illness. The famous case reports of the first human heart transplant by Christian Barnard (1967) [7], speech impairment in patients with left hemispheric brain lesions published by Paul Broca (1861) [8], and the teratogenicity of a multitude of compounds are some of many illustrious examples. These crucial observations and records stimulate the flow of medical and scientific knowledge among clinicians and researchers so as to improve the effective and safety of medical practice and arouse awareness, inquiry, and further investigation.

The use of case reports in medical education to train the future cadre of medical professionals is well-documented[9]. Case reports in *BMC Research Notes* will provide a home in which to document different approaches to the evaluation, diagnosis, treatment, and long-term management of common clinical conditions as well as rare case presentations and diseases. These cases can be discussed at grand rounds to foster dialogue and discussion among senior healthcare professionals with those in training to learn of different clinical case experiences. While RCTs provide one to two limited key messages from the outcomes of the study, case reports now offered by the *Journal* document the full picture of clinical care on a case-by-case basis.

The *BMC Series* medical journals (http://www.biomedcentral.com/authors/bmcseries) already publish case reports that contribute to the dissemination of medical knowledge or draw attention to the need for change in clinical practice or diagnostic/prognostic approaches. BioMed Central also publishes a specific case reports journal*, Journal of Medical Case Reports* (http://www.jmedicalcasereports.com/about), which has similar criteria for publication to those of the *BMC Series*. In keeping with the inclusive ethos of *BMC Research Notes*, it is envisioned that well-reported, ethically and scientifically sound, peer-reviewed case reports which may not meet these criteria will also have educational value.

Continuing *BMC Research Notes*’ reputation for innovation, a new model for peer review of case report articles will also be introduced. The nature of case reports implies that “further experiments” are never possible and “additional analyses needed” are uncommon. In light of this, case reports submitted to the journal will be reviewed by a single specialist Associate Editor, who will provide a report and make a decision. We anticipate that this will enable rapid decision making whilst still providing a thorough peer review process, and make for a smooth publication experience for authors.

## Discussion

As thoroughly contended, case reports serve a unique purpose in the scientific and medical community by offering anecdotal evidence where experimental evidence is lacking. It provides a means to disseminate medical knowledge from high-practicing individuals in an intellectually appealing fashion. Be they reports of new diseases, instances of unforeseen though crucial patient variations, records of unintended adverse reactions, or elegantly-written synopses of common diseases, case reports serve the purpose of aiding young health professionals that seek to gain basic clinical knowledge and insight into the approach of a patient as well as the experienced physician that encounters a bizarre case in his or her daily practice. Countless medical diseases, syndromes, and drugs—HIV/AIDS, Broca’s aphasia, multiple myeloma, psychiatric disorders, Peutz-Jeghers syndrome, teratogens, penicillin, etc.—were discovered by initial publishing of case reports followed by the recognition of these reported phenomena in the general medical and scientific community [8,10-13]. Though it is quixotic to expect similar events, *BMC Research Notes* strives to contribute to this process and advance the fields of science and medicine through one brief patient note at a time.

## Abbreviations

AIDS: Acquired immune deficiency syndrome; BC: Before Christ; HIV: Human immunodeficiency virus; RCT: Randomized Clinical Trial.

## Competing interests

AJCM serves as Associate Editor for BMC Research Notes. WFGB declares that he has no competing interests.

## Authors’ contributions

AJCM and WFGB were equal contributors in writing the manuscript. Both read and approved the final version of the manuscript. All authors read and approved the final manuscript.
